# Prevalence and Pattern of Personality Disorders and Psychiatric Comorbidities Among Patients With Substance Use Disorders in a Tertiary Care De-addiction Setting: A Cross-Sectional Study

**DOI:** 10.7759/cureus.114374

**Published:** 2026-08-11

**Authors:** Lalita Sunda, Bhavneesh Saini, Amit Jain, Pir D Bansal, Jasjit Kaur, Milanjot Kaur

**Affiliations:** 1 Department of Psychiatry, Guru Gobind Singh Medical College and Hospital, Faridkot, IND; 2 Department of Pharmacology, Guru Gobind Singh Medical College and Hospital, Faridkot, IND

**Keywords:** cluster b personality disorders, international personality disorder examination (ipde), personality disorders, psychiatric comorbidity, substance use disorder

## Abstract

Background

Substance use disorders (SUDs) frequently coexist with personality disorders (PDs), contributing to poorer treatment adherence, relapse, and clinical outcomes. Their coexistence with other psychiatric comorbidities further complicates management and highlights the need for comprehensive assessment. However, data regarding the prevalence and pattern of PDs among patients with SUDs in Indian tertiary care settings remain limited. This study aimed to determine the prevalence and pattern of PDs and personality traits among patients with SUDs, evaluate associated psychiatric comorbidities, and examine their relationship with different types of substance use.

Methods

A hospital-based cross-sectional study was conducted in the Department of Psychiatry, Guru Gobind Singh Medical College and Hospital, Faridkot, Punjab, over a nine-month period from 01 October 2025 to 30 June 2026. A total of 200 consecutive patients aged 18 to 60 years who fulfilled the Diagnostic and Statistical Manual of Mental Disorders, Fifth Edition (DSM 5) criteria for SUD and were admitted to the de-addiction ward were enrolled. Sociodemographic and clinical information was collected using a structured psychiatric interview proforma. PDs and personality traits were assessed using the International Personality Disorder Examination (IPDE). Psychiatric comorbidities were evaluated through detailed clinical interviews, mental status examination, and standardized rating scales including the Hamilton Anxiety Rating Scale, Hamilton Depression Rating Scale, Yale Brown Obsessive Compulsive Scale, and Young Mania Rating Scale, whenever clinically indicated.

Results

The mean age of the participants was 30.55 ± 6.40 years. Alcohol and opioid use were the most common substances, each accounting for 30.0% of cases, followed by multiple substance use (24.0%) and cannabis use (16.0%). Personality traits were identified in 27.5% of participants, while 20.5% met the diagnostic criteria for a PD. Dissocial and impulsive PDs were the most prevalent, each observed in 5.5% of participants. Cluster B PDs constituted the largest group (12.5%), followed by Cluster C (4.5%) and Cluster A (3.5%). Psychiatric comorbidity was present in 6.5% of participants, with depression (3.0%) being the most frequent, followed by anxiety (2.0%), obsessive-compulsive disorder (1.0%), and mania (0.5%). No statistically significant association was found between the type of substance used and the presence of PD (χ² = 1.069, p = 0.785). No statistically significant association was observed between substance type and PD clusters using the Fisher-Freeman-Halton exact test (exact two-sided p = 0.976).

Conclusion

PDs were present in one-fifth of patients with SUDs, with dissocial and impulsive PDs being the most frequently observed individual diagnoses. Cluster B was the most frequently observed PD cluster in this inpatient SUD population. No significant association was found between the type of substance used and either the presence of PD or the distribution of PD clusters. Routine assessment of personality pathology and psychiatric comorbidities may support individualized clinical management.

## Introduction

Personality refers to enduring patterns of thoughts, emotions, and behaviours that characterize an individual. Gordon Allport described personality as a dynamic organization of psychophysical systems that determine characteristic behaviour and thought [1]. Similarly, Funder defined personality as characteristic patterns of thought, emotion, and behaviour along with the psychological mechanisms underlying them [2]. Personality traits represent stable dimensions of individual differences in thinking, feeling, and behaviour [3].

Personality development is influenced by the interaction of biological, genetic, and environmental factors. Early temperamental traits play an important role in shaping personality across the lifespan. Four temperamental dimensions - harm avoidance, novelty seeking, reward dependence, and persistence - are considered particularly influential in this process [4]. Personality traits are generally measured along a continuum. The widely accepted Five Factor Model describes personality through five major traits: extraversion, agreeableness, conscientiousness, neuroticism, and openness to experience [3].

Developmental and environmental factors may also contribute to neuropsychiatric vulnerability across the life course. Early life stress, family environment, social adversity, and exposure to potentially harmful substances during critical developmental periods may influence emotional regulation, cognition, and behavioural patterns. Emerging literature has also examined possible associations between prenatal nicotine exposure and later neurodevelopmental outcomes. Although such findings provide a broader developmental framework for understanding psychiatric vulnerability, they should not be interpreted as direct evidence for personality disorders (PDs) or substance use disorders (SUDs) [5].

PDs are maladaptive and inflexible patterns of personality traits that cause significant distress or impairment in functioning. According to the Diagnostic and Statistical Manual of Mental Disorders (DSM-5), PDs are categorized into three clusters [6]. Cluster A includes paranoid, schizoid, and schizotypal PDs characterized by odd or eccentric behaviour. Cluster B includes antisocial, borderline, histrionic, and narcissistic PDs associated with dramatic, emotional, or erratic behaviour. Cluster C includes avoidant, dependent, and obsessive-compulsive PDs characterized by anxious or fearful behaviour [7].

The epidemiology of PDs is less clearly understood due to challenges in their assessment in population surveys. Prevalence rates range from 4% to 15% depending on study design and population [8]. A multinational study reported a point prevalence of 6.1%, with higher rates in North and South America and lower rates in Europe [9]. PDs are more frequently observed among individuals seeking psychiatric care and those involved in the criminal justice system [10]. Nearly half of psychiatric patients and more than two-thirds of individuals in the criminal justice system have been reported to have one or more PDs [11].

SUDs involve the harmful use of psychoactive substances such as alcohol, narcotics, and other addictive drugs. These substances affect the brain by altering neurotransmitter systems, particularly dopamine pathways involved in reward and motivation. Repeated stimulation of these pathways produces pleasurable effects and reinforces continued substance use, increasing the risk of addiction [12]. The risk of substance abuse is influenced by a combination of biological, psychological, and environmental factors.

PDs frequently co-occur with SUDs, complicating diagnosis and treatment. Traits such as impulsivity, emotional instability, and poor stress tolerance may increase vulnerability to substance abuse, while chronic substance use may exacerbate underlying personality pathology. Indian studies have reported varying prevalence rates of PDs among substance users, with higher rates among inpatients (21.55%) compared to outpatients (1.07%), while international studies report prevalence around 14.5% [13].

SUDs are also associated with psychiatric comorbidities, including mood, anxiety, and psychotic disorders [14]. Antisocial and borderline PDs have been reported among individuals using heroin and cocaine, while polysubstance use may be associated with greater personality disturbance [15]. The coexistence of these conditions may contribute to greater clinical complexity and poorer treatment outcomes.

Despite the established relationship between SUD, personality pathology, and psychiatric comorbidity, evidence describing these patterns among patients receiving inpatient de-addiction treatment in Indian tertiary care settings remains limited. Differences in substance use patterns, sociodemographic characteristics, treatment settings, and clinical presentation may influence the distribution of personality pathology and psychiatric comorbidities across populations. Therefore, the present study was undertaken to assess the distribution of personality traits and PDs, their cluster patterns, and psychiatric comorbidities among patients with SUD admitted to an inpatient de-addiction service at a tertiary care centre in India. The findings may provide clinically relevant information for comprehensive assessment and individualized management of this population.

The primary objective of the study was to assess the prevalence of PDs among individuals with SUDs admitted to an inpatient de-addiction service at a tertiary care centre. The secondary objectives were (a) to describe the distribution of PD clusters and individual PD categories in the study population, (b) to assess the distribution of psychiatric comorbidities among individuals with SUDs, and (c) to examine the relationship between the type of substance used and the presence and distribution of PDs.

## Materials and methods

Study design and population

The present cross-sectional study was conducted in the Department of Psychiatry at Guru Gobind Singh Medical College and Hospital (GGSMC), Faridkot, Punjab, a tertiary care teaching hospital with specialized de-addiction services, after obtaining approval from the Institutional Ethics Committee (Approval No. BFUHS/2K26p-TH/18/18, dated 18 September 2025). The study included patients aged 18 to 60 years diagnosed with SUD who were admitted to the de-addiction ward. The study was conducted over a nine-month period from 01 October 2025 to 30 June 2026, during which eligible patients were screened and enrolled after obtaining written informed consent.

Inclusion and exclusion criteria

Participants aged 18-60 years who were diagnosed with SUD according to the Diagnostic and Statistical Manual of Mental Disorders, Fifth Edition (DSM-5) [16], and who provided informed consent were included in the study. Patients were excluded if they had a history of neurological illnesses such as brain tumors, stroke, or space-occupying lesions; major or unstable medical illnesses including hepatic, renal, gastroenterological, respiratory, cardiovascular (including ischemic heart disease), endocrinologic, oncologic, immunologic, or hematologic disorders; intellectual disability; or substance-induced psychiatric disorders (F1x.5).

Sample size and sampling technique

The sample size was calculated using the pooled prevalence of any PD reported by Winsper et al. [17], who reported a global prevalence of 7.8% in a systematic review and meta-analysis of community populations. Using the formula n = (Z² × P × [1 − P]) / d², where Z = 1.96 for a 95% confidence level, P = 0.078, and d = 0.05, the calculated minimum sample size was approximately 111 participants. An absolute precision of 0.05 was selected to estimate the prevalence within ±5 percentage points of the expected prevalence. Although the prevalence estimate was derived from community populations and was not specific to patients with SUDs, it was selected because it directly represented the prevalence of diagnosed PDs, which was the primary outcome of the present study. Finite population correction was not applied because the underlying inpatient SUD population was not a defined finite population from which the study sample was drawn. To improve precision and allow for possible incomplete data, 200 eligible participants were ultimately enrolled. Consecutive non-probability sampling was used, and eligible patients admitted to the de-addiction ward during the study period were consecutively enrolled

Study procedure and data collection

After obtaining written informed consent, sociodemographic and clinical information was recorded using a structured psychiatric thesis/interview proforma. Patients fulfilling the DSM-5 diagnostic criteria for SUD were enrolled [16]. PDs and personality traits were assessed using the International Personality Disorder Examination (IPDE), a semi-structured interview comprising 67 items that evaluate domains including work, self, interpersonal relationships, affect, reality testing, and impulse control [18]. The IPDE was administered by the treating psychiatrist using the standardized interview format; however, formal details of assessor training and inter-rater standardization were not prospectively documented.

The IPDE was used as the instrument for assessment of personality pathology. DSM-5 was used for the diagnosis of SUD, whereas personality pathology was assessed using the IPDE framework. The IPDE categories were subsequently grouped into the conventional Cluster A, B and C categories for analysis. Paranoid and schizoid categories were grouped under Cluster A; dissocial, impulsive, borderline and histrionic categories under Cluster B; and anankastic, dependent and anxious/avoidant categories under Cluster C. Thus, the cluster classification was used for analytical presentation of IPDE findings and did not imply that the IPDE itself was a DSM-5 diagnostic instrument.

The exact duration of abstinence before IPDE assessment was not systematically recorded. Assessment was performed during the inpatient de-addiction stay after clinical evaluation; however, the possibility that early abstinence or withdrawal-related symptoms may have influenced personality assessment cannot be completely excluded.

Psychiatric comorbidities were assessed through detailed psychiatric interviews and mental status examination during the inpatient stay. Psychiatric diagnoses were established clinically by the treating psychiatrist according to DSM-5 diagnostic criteria. When clinically indicated, symptom severity was additionally assessed using the Hamilton Anxiety Rating Scale (HAM A) [19], Hamilton Depression Rating Scale (HDRS) [20], Yale Brown Obsessive Compulsive Scale (Y BOCS) [21], and Young Mania Rating Scale (YMRS) [22]. These scales were not administered universally to all participants and were used to characterize symptom severity rather than as standalone diagnostic tools. Therefore, scale-based symptom thresholds were not used as the sole basis for diagnosing psychiatric comorbidities.

Statistical analysis

Data were entered into Microsoft Excel (Redmond, WA, USA) and analyzed using IBM SPSS Statistics for Windows, Version 27.0 (IBM Corp., Armonk, NY, USA). Categorical variables were summarized as frequencies and percentages, while continuous variables were summarized using appropriate measures of central tendency and dispersion. Associations between categorical variables were assessed using Pearson chi-square tests when assumptions were satisfied. Expected cell frequencies were examined for all chi-square analyses, and exact tests were used when expected frequencies were insufficient. For the 4 × 4 comparison between substance type and personality disorder cluster, the Fisher-Freeman-Halton exact test was used because nine of 16 cells had expected counts below 5. All 200 participants had complete data for the primary study variables, and no imputation of missing data was required. The analyses were primarily descriptive and unadjusted. Multivariable regression analysis was not performed because of the limited number of participants with PDs and sparse distribution across specific PD categories, which could result in model instability and overfitting. A two-tailed p-value < 0.05 was considered statistically significant.

## Results

The mean age of the participants was 30.55 ± 6.40 years, indicating that most participants were young adults. Half of the participants belonged to nuclear families (50.0%), accounting for 100 participants, followed by 65 participants from joint families (32.5%) and 35 from three-generation families (17.5%). Most participants were married (67.0%), comprising 134 individuals, while 66 participants were unmarried (33.0%). Regarding occupation, 76 participants were self-employed (38.0%), 58 were skilled or semi-skilled workers (29.0%), 40 were unemployed (20.0%), and 26 were engaged in unskilled work (13.0%). Overall, the study population predominantly consisted of young, married individuals from nuclear families, with self-employment being the most common occupation (Table 1).

**Table 1:** Sociodemographic Characteristics of the Study Participants (n = 200).

Variable	Category	n (%)
Age (years)	Mean ± SD	30.55 ± 6.40
Family Type	Nuclear	100 (50.0)
	Joint	65 (32.5)
	Three-generation	35 (17.5)
Marital Status	Married	134 (67.0)
	Unmarried	66 (33.0)
Occupation	Self-employed	76 (38.0)
	Skilled/Semi-skilled	58 (29.0)
	Unskilled	26 (13.0)
	Unemployed	40 (20.0)

Alcohol was the most commonly used substance, reported by 60 participants (30.0%), followed closely by opioid use in 60 participants (30.0%). Multiple substance use was observed in 48 participants (24.0%), while cannabis use was reported by 32 participants (16.0%). Overall, alcohol and opioid use were equally the most prevalent forms of substance use in the study population (Table 2).

**Table 2:** Distribution of Substance Use among the Study Participants (n = 200).

Category	n (%)
Alcohol	60 (30.0)
Opioid	60 (30.0)
Cannabis	32 (16.0)
Multiple substances	48 (24.0)

Personality traits were identified in 55 participants (27.5%), while 145 participants (72.5%) did not exhibit any significant personality traits. PDs were diagnosed in 41 participants (20.5%), whereas 159 participants (79.5%) did not meet the diagnostic criteria for any PD. Overall, approximately one-fourth of the participants had personality traits, and one-fifth had a diagnosable PD (Table 3).

**Table 3:** Prevalence of Personality Traits and Personality Disorders among the Study Participants (n = 200).

Variable	Category	n (%)
Personality Trait	Present	55 (27.5)
	Absent	145 (72.5)
Personality Disorder	Present	41 (20.5)
	Absent	159 (79.5)

Among personality traits, 12 participants had dissocial traits (6.0%) and 11 had impulsive traits (5.5%), making these the most common personality traits. Anankastic traits were observed in seven participants (3.5%), followed by schizoid traits in six (3.0%). Dependent and paranoid traits were each present in five participants (2.5%), while anxious (avoidant) traits were identified in four (2.0%). Histrionic traits were seen in three participants (1.5%), and borderline traits in two (1.0%). Among PDs, dissocial and impulsive PDs were the most common, each diagnosed in 11 participants (5.5%). Schizoid PD was present in five participants (2.5%), while anankastic and dependent PDs were each identified in four (2.0%). Paranoid and borderline PDs were each diagnosed in two participants (1.0%), whereas histrionic and anxious (avoidant) PDs were the least common, each affecting one participant (0.5%) (Table 4).

**Table 4:** Distribution of Personality Traits and Personality Disorders by Type According to IPDE (n = 200).

Type	Personality Trait n (%)	Personality Disorder n (%)
Dissocial	12 (6.0)	11 (5.5)
Impulsive	11 (5.5)	11 (5.5)
Schizoid	6 (3.0)	5 (2.5)
Anankastic	7 (3.5)	4 (2.0)
Dependent	5 (2.5)	4 (2.0)
Paranoid	5 (2.5)	2 (1.0)
Borderline	2 (1.0)	2 (1.0)
Histrionic	3 (1.5)	1 (0.5)
Anxious (Avoidant)	4 (2.0)	1 (0.5)

Cluster B PDs were the most common, affecting 25 participants (12.5%). Cluster C PDs were identified in nine participants (4.5%), while Cluster A PDs were present in seven participants (3.5%). The majority of the study population, 159 participants (79.5%), did not meet the criteria for any PD. Psychiatric comorbidity was observed in 13 participants (6.5%) (Table 5).

**Table 5:** Distribution of Personality Disorder Clusters and Psychiatric Comorbidity among the Study Participants (n = 200).

Variable	n (%)
Cluster A	7 (3.5)
Cluster B	25 (12.5)
Cluster C	9 (4.5)
No Personality Disorder	159 (79.5)
Presence of Psychiatric Comorbidity	13 (6.5)

Personality disorder was identified in 15 opioid users (25.0%), 11 alcohol users (18.3%), 6 cannabis users (18.8%), and 9 multiple substance users (18.8%). The majority of participants in each substance use group did not have a PD, including 49 alcohol users (81.7%), 45 opioid users (75.0%), 26 cannabis users (81.2%), and 39 multiple substance users (81.2%). Although PDs were relatively more common among opioid users, there was no statistically significant association between the type of substance used and the presence of a PD (χ² = 1.069, p = 0.785) (Table 6).

**Table 6:** Association Between Type of Substance Use and Presence of Personality Disorder (PD) (n = 200). Chi-square test was used for comparison. A p value <0.05 was considered statistically significant.

Type of Substance	PD Present n (%)	PD Absent n (%)	Statistics
Alcohol (n=60)	11 (18.3%)	49 (81.7%)	χ² = 1.069; p = 0.785
Opioid (n=60)	15 (25.0%)	45 (75.0%)	
Cannabis (n=32)	6 (18.8%)	26 (81.2%)	
Multiple (n=48)	9 (18.8%)	39 (81.2%)	

Most participants across all substance use groups did not have a PD, including 49 alcohol users (81.7%), 45 opioid users (75.0%), 26 cannabis users (81.2%), and 39 multiple substance users (81.2%). Among those with PDs, Cluster B was the most common, affecting six alcohol users (10.0%), nine opioid users (15.0%), three cannabis users (9.4%), and seven multiple substance users (14.6%). Cluster A and Cluster C PDs were less frequently observed across all substance categories. However, there was no statistically significant association between the type of substance used and the distribution of PD clusters (Fisher-Freeman-Halton exact test = 3.157, exact two-sided p = 0.976) (Table 7).

**Table 7:** Association Between Type of Substance Use and Personality Disorder (PD) Clusters (n = 200). Fisher-Freeman-Halton exact test was used for comparison. A p-value <0.05 was considered statistically significant.

Type of Substance	No PD n (%)	Cluster A n (%)	Cluster B n (%)	Cluster C n (%)	Statistics
Alcohol (n=60)	49 (81.7%)	2 (3.3%)	6 (10.0%)	3 (5.0%)	Fisher-Freeman-Halton exact test = 3.157; Exact two-sided p = 0.976; Cramer's V = 0.119
Opioid (n=60)	45 (75.0%)	3 (5.0%)	9 (15.0%)	3 (5.0%)	
Cannabis (n=32)	26 (81.2%)	1 (3.1%)	3 (9.4%)	2 (6.3%)	
Multiple (n=48)	39 (81.2%)	1 (2.1%)	7 (14.6%)	1 (2.1%)	

Dissocial PD was identified in six opioid users (10.0%), two multiple substance users (4.2%), two alcohol users (3.3%), and one cannabis user (3.1%). Although dissocial PD was more frequently observed among opioid users, the association between substance type and dissocial PD was not statistically significant (χ² = 6.958, p = 0.073; Cramer's V = 0.187) (Table 8).

**Table 8:** Association Between Substance Type and Dissocial Personality Disorder (PD) (n = 200). Chi-square test was used for comparison. A p value <0.05 was considered statistically significant.

Substance Type	Dissocial PD Present n (%)	Statistics
Alcohol	2 (3.3%)	χ² = 6.958; p = 0.073; Cramer's V = 0.187
Opioid	6 (10.0%)	
Cannabis	1 (3.1%)	
Multiple	2 (4.2%)	

## Discussion

The present cross-sectional study was conducted in the Department of Psychiatry at Guru Gobind Singh Medical College and Hospital, Faridkot, over a period of nine months. A total of 200 patients aged 18-60 years diagnosed with SUDs according to DSM-5 criteria and admitted to the de-addiction ward were included using consecutive non-probability sampling. PDs and traits were assessed using the IPDE, while psychiatric comorbidities were evaluated through clinical interviews and standardized rating scales. The study aimed to estimate the prevalence and pattern of PDs and personality traits among individuals with SUDs and to examine their association with different types of substance abuse.

In the present study, the mean age of participants was 30.55 ± 6.40 years, indicating that the study population largely comprised young adults. Half of the participants belonged to nuclear families (50.0%), followed by joint families (32.5%) and three-generation families (17.5%). Most participants were married (67.0%), and self-employment was the most common occupation (38.0%).

These findings are comparable with previous studies reporting that SUDs predominantly affect young adults. Rawal et al. reported that most patients with SUDs were aged between 18-39 years, with the highest proportion in the 30-39 year age group [23]. Hasan et al. also reported a comparable mean age of 31.1 ± 9.3 years among patients with substance dependence [15].

However, some differences have been reported in other studies. Rawal et al. observed that the majority of participants lived in joint families [23], whereas nuclear families were more common in the present study. Casadio et al. reported an older study population with a mean age of 40.9 ± 10.8 years, indicating possible variations across different study settings [24]. González et al. also reported no significant association between most sociodemographic variables and PDs, which is consistent with the present findings [25]. Langås et al. reported that patients with PDs had a lower mean age and poorer occupational functioning compared with those without PDs [26].

In the present study, personality traits were identified in 27.5% of participants. Dissocial personality trait (6.0%) and impulsive trait (5.5%) were the most frequently observed, followed by anankastic (3.5%) and schizoid traits (3.0%). Other traits included dependent (2.5%), paranoid (2.5%), anxious (avoidant) (2.0%), histrionic (1.5%), and borderline traits (1.0%). In the present study, 20.5% of participants were diagnosed with a PD. Dissocial and impulsive PDs were the most common (5.5% each), followed by schizoid (2.5%), dependent (2.0%), and anankastic PDs (2.0%). Cluster-wise distribution showed that Cluster B PDs were the most prevalent (12.5%), followed by Cluster C (4.5%) and Cluster A (3.5%).

These findings are consistent with several previous studies that have reported a predominance of Cluster B PDs among individuals with SUDs. Casadio et al. reported Cluster B PDs in 32.8% of participants, emphasizing the association between impulsivity and substance use [24]. Langås et al. also reported that antisocial and borderline PDs were among the most common PDs in this population [26].

However, some studies have reported higher overall prevalence rates of PDs. Rawal et al. reported that 63% of patients had at least one PD [23]. González et al., however, reported a somewhat different pattern, with Cluster C PDs being the most prevalent [25]. These differences may be related to variations in study design, diagnostic methods, and study populations.

Psychiatric comorbidity was observed in 6.5% of participants in the present study. Depression was the most common comorbid condition (3.0%), followed by anxiety (2.0%), obsessive-compulsive disorder (1.0%), and mania (0.5%). The available psychiatric rating scale findings indicated low symptom severity among participants assessed with the respective scales.

These findings contrast with several previous studies reporting higher psychiatric comorbidity among patients with SUDs. Rawal et al. reported anxiety disorders as the most frequent psychiatric diagnosis (61%) [23]. Casadio et al. also reported Axis I psychiatric disorders in approximately 12% of patients [24]. Similarly, Langås et al. found that a large proportion of patients had at least one Axis I psychiatric disorder, particularly anxiety disorders [26]. The lower prevalence observed in the present study may be related to the inpatient detoxification setting and stabilization of acute psychiatric symptoms.

In the present study, alcohol and opioid use were the most common substances (30.0% each), followed by multiple substance use (24.0%) and cannabis use (16.0%). PD was present in 25.0% of opioid users, 18.3% of alcohol users, 18.8% of cannabis users, and 18.8% of multiple substance users, with no statistically significant association between substance type and PD (χ² = 1.069, p = 0.785).

These findings are consistent with González et al., who also reported no significant association between the type of substance used and the presence of PDs [25]. In the present study, Cluster B disorders were the most frequently observed PD cluster across substance categories. However, there was no statistically significant association between substance type and the distribution of PD clusters (Fisher-Freeman-Halton exact test = 3.157, exact two-sided p = 0.976). However, Hasan et al. reported significant associations between specific PDs and particular substances, particularly antisocial PD with opioid use [15].

The relationship between substance use and psychiatric disorders is likely influenced by interacting biological, psychological, cognitive, and environmental mechanisms. Emerging technology-assisted approaches, including artificial intelligence-based assessment of stress, anxiety, and mental health, may support future psychiatric screening and monitoring, although such approaches require further validation before routine clinical use [27]. Recent work on peripheral biomarkers such as phosphorylated tau and amyloid β, as well as emerging therapeutic approaches involving modern probiotics, illustrates the broader movement toward biologically informed approaches to neuropsychiatric disorders [28,29]. These developments provide a broader conceptual framework but should not be interpreted as direct evidence for PDs in patients with SUD.

Personality and neuropsychiatric vulnerability may also be influenced by developmental experiences and environmental exposures across the life course. Early life stressors, family environment, social adversity, and exposure to potentially harmful substances during critical developmental periods may influence emotional regulation, cognition, and behavioural patterns. Recent literature has examined possible links between prenatal nicotine exposure and later neurodevelopmental outcomes, highlighting the importance of considering developmental and environmental determinants in neuropsychiatric research [5]. However, such emerging evidence should be interpreted cautiously and does not establish a direct relationship with PDs or SUD.

The present study provides insights into the prevalence and pattern of PDs among individuals with SUDs in a tertiary care setting. The use of a structured diagnostic instrument (IPDE) and standardized psychiatric rating scales enhanced the consistency of clinical assessment. However, several limitations should be considered. The study was conducted at a single tertiary care center and included an inpatient-only population, which may limit the generalizability of the findings to patients receiving treatment in other settings. Consecutive non-probability sampling may also have introduced selection bias. The cross-sectional design prevents assessment of temporal relationships and does not allow causal inferences regarding the relationship between substance use and PDs. The exact duration of abstinence before IPDE assessment was not systematically recorded; therefore, the potential influence of early abstinence or withdrawal-related symptoms on personality assessment cannot be completely excluded.

Formal documentation of assessor training and inter-rater standardization for IPDE administration was not available, which may have introduced some degree of interviewer-related variability. Psychiatric rating scales were administered based on clinical indication rather than systematically to all participants; therefore, some psychiatric comorbidities, particularly those with less prominent symptoms, may have been underdetected. The relatively small number of participants with PDs and the sparse distribution across specific PD categories precluded reliable multivariable adjustment. Therefore, the observed findings should be interpreted as unadjusted associations and should not be considered independent or causal relationships. Future multicenter studies with larger samples, standardized assessor training, systematic psychiatric assessment, and longitudinal designs are needed to better understand the temporal relationship between personality pathology and SUDs.

## Conclusions

The present study showed that a considerable proportion of patients with substance use disorders exhibited personality traits and personality disorders. Dissocial and impulsive personality disorders were the most common, with Cluster B disorders being the predominant cluster. Psychiatric comorbidities were observed in a small proportion of participants, with depression being the most frequent, followed by anxiety, obsessive-compulsive disorder, and mania. No significant association was found between the type of substance used and either the presence of personality disorder or the distribution of personality disorder clusters. These findings highlight the importance of routine assessment of personality pathology and psychiatric comorbidities in individuals with substance use disorders for better clinical management.
